# Evaluating undercounts in epidemics: Response to Maruotti et al. (2022)

**DOI:** 10.1002/jmv.28474

**Published:** 2023-01-26

**Authors:** Michael Li, Jonathan Dushoff, David J. D. Earn, Benjamin M. Bolker

**Affiliations:** ^1^ Public Health Risk Science Division, National Microbiology Laboratory Public Health Agency of Canada Guelph Ontario Canada; ^2^ Department of Mathematics & Statistics McMaster University Hamilton Ontario Canada; ^3^ Department of Biology McMaster University Hamilton Ontario Canada

## 1 INTRODUCTION

Several papers^1^, ^2^, ^3^ have promoted formulas that claim to provide bounds on the completeness of sampling of infectious disease cases, based only on case reports. We believe these approaches are fundamentally flawed, and that it is impossible to estimate undercounting from incidence data without a specialized sampling design or some kind of auxiliary information.

The authors use mark‐recapture formulas developed by Chao^4^ and others^5^ to estimate bounds on true population sizes based on the numbers of individuals observed multiple times. For example, the proposed estimator for the lower bound on unobserved individuals (hidden cases) is,

$$\hat{H}\left(t\right)=\Delta N\left(t\right)\frac{\Delta N\left(t\right)-1}{\Delta N\left(t-1\right)+1},$$

where ΔN(t) is the number of new cases observed per reporting period; extended formulas adjust for mortality and recovery. The upper bound^1^, ^3^ also involves ΔN(t−2).

## 2 CRITIQUE

### 2.1 Conceptual argument

This approach misuses the mark‐recapture formulas. Cases identified at time t−1 are claimed to be representative of the number of cases counted twice: why? The fact that the same individual *could* be counted twice in the cumulative case report (for some sampling designs) is irrelevant. How can comparing yesterday's count to today's provide information about the completeness of sampling?

In principle, the number of unobserved (hidden) cases could be estimated if cases can be reidentified, or even with unmarked/unidentified cases given an appropriate sampling design.^6^ In practice public health case reporting rarely uses such sampling designs. Case reporting is usually exclusive (i.e., someone who has been identified as a case will not be reported again later), or anonymized so that we cannot identify which particular individuals are double‐counted because they are infected, and sampled, in two different case‐reporting instances. Mark‐recapture methods can provide valuable public health information in specific scenarios such as contact‐tracing studies, but “one needs at least two sources of information with individual case reporting and a unique personal identifier for each case”.^7^ This limitation is fundamental to mark‐recapture methods; standard case‐reporting time series, which do not identifiably resample the same individuals, provide no information with which we could estimate the fraction of the population observed.

### 2.2 Mathematical argument

The simplest mathematical illustration of the problems with the method occurs during the exponential‐growth phase of the epidemic (when the authors have suggested that their method is most appropriate). During this phase, the incidence (true number of new infections: I(t)) grows at a rate λ per time step, that is, $I\left(t\right)=I_{0}\lambda^{t}$. Suppose a fraction a (the *ascertainment ratio*) of these cases is reported (i.e., a is the ratio of reported cases to the true incidence; for simplicity, we assume here that a is constant over time). An estimated lower bound on the number of hidden cases, $\hat{H}$ can be converted to an upper bound on the estimated ascertainment ratio, $\hat{a}$. Using the simpler, non‐bias‐corrected formula:

$$\begin{array}{lll} \hat{a} & = & \frac{\;\text{observed cases}}{\text{observed cases}+\text{hidden cases}\;}\\ & = & \frac{\Delta N\left(t\right)}{\Delta N\left(t\right)+\hat{H}\left(t\right)}\\ & = & \frac{\Delta N\left(t\right)}{\Delta N\left(t\right)+\frac{{\left[\Delta N\left(t\right)\right]}^{2}}{\Delta N\left(t-1\right)}}\\ & = & {\left(1+\frac{\Delta N\left(t\right)}{\Delta N\left(t-1\right)}\right)}^{-1}\\ & = & {\left(1+\frac{aI_{0}\lambda^{t}}{aI_{0}\lambda^{t-1}}\right)}^{-1}\\ & = & \frac{1}{1+\lambda }. \end{array}$$

The estimated upper bound of the ascertainment ratio $\hat{a}$ thus depends only on the epidemic growth rate; it is independent of the true ascertainment ratio. Furthermore, epidemics typically grow at rates of a few percent per reporting period; an epidemic with more than 20% growth per reporting period (λ>1.2) would be catastrophic. Thus, the upper bound on the ascertainment ratio during the exponential phase would typically range only from about 0.45–0.5.

Applying a bias correction decreases the lower bound on the number of hidden cases, thus increasing the upper bound on $\hat{a}$. The results also depend on the overall number of reported cases, so the pattern is more complicated, but as we show below the estimated upper and lower bounds are still largely independent of the true ascertainment ratio.

### 2.3 Simulation example

We ran simulations using a Richards curve for the cumulative incidence of the epidemic^8^:

(1) $$\;\text{Cumulative incidence}\;\left(t\right)=\frac{K}{{\left(1+se^{-sr\left(t-h\right)}\right)}^{1∕s}},$$

where K is the final size of the epidemic, h is the time of inflection, and r is the initial growth rate. The Richards curve is a widely used phenomenological model for epidemic curves^9^, ^10^ and, according to the authors, is the same method they used to test their approach (pers. comm.). We computed expected incidence by differencing the cumulative incidence,^8^ drew a random negative binomial deviate with mean equal to the expected incidence, and used a binomial sample with probability equal to the ascertainment ratio a to get the number of observed cases. Throughout, we used a shape parameter of s=2 and a final epidemic size of $10^{5}$ for the Richards curve, and a negative binomial dispersion parameter k=5. We varied the reporting period (Δt={1,7}); initial incidence ($I_{0}=\left\{20,40\right\}$); epidemic growth rate (*r* = 0.01 to 0.08 per day); and ascertainment ratio (a = 0.05 to 0.6). (We solved the Richards equation numerically to recover the h parameter given a value of the initial incidence, which is the derivative of Equation (1) at time zero.) These ranges encompass typical parameters of epidemic outbreaks (SARS‐CoV‐1, COVID‐19, monkeypox, etc.), but we argue that the precise numerical values are not very important. The key aspects of a simulation are the epidemic growth rate (λ=exp(rΔt)), which is the primary determinant of the ascertainment ratio bounds computed according to Maruotti et al's^2^ method, and the typical number of cases reported per period, which determines the effects of the bias correction terms.

We ran each simulation for 100 days and used the R package asymptor ^11^ to compute bounds on the ascertainment ratio.

The authors indicated (pers. comm.) that they intended the estimator to be used at the beginning of an epidemic. Therefore we considered only sample points when the number of cases was between 5 and 500 (exclusive) and the lower bound estimator for hidden cases was greater than 1.

**Figure 1:**
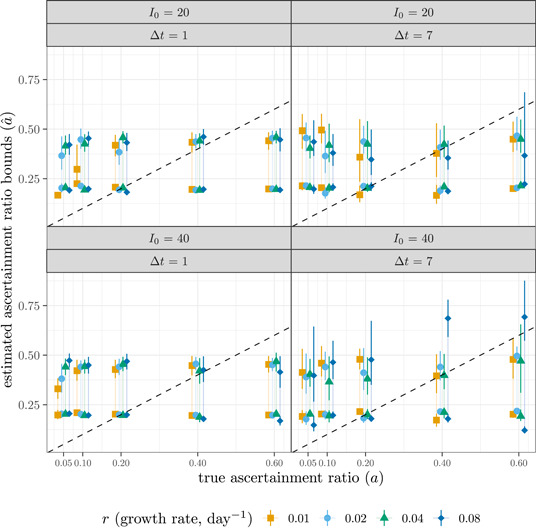
Comparison of true ascertainment ratio (a) to estimated lower and upper bounds of ascertainment ratio ($\hat{a}$). Dashed line is the one‐to‐one line (estimated = true).

For each simulation run (80 in total), we computed the mean and confidence intervals for the estimated lower and upper bounds of $\hat{a}$ over time (Figure 1). The bounds on $\hat{a}$ rarely overlap the true value, and are *largely independent of the true values of* a. The only noticeable signal arises from the bias‐correction terms: simulations with lower overall case numbers (low r, low a,Δt=1) have larger lower bounds and smaller upper bounds. The relationship between $\hat{a}$ and the growth rate r is barely visible as increasing values of the upper bound with r for the cases with Δt=1 and low true ascertainment ratio; otherwise, this pattern is swamped by the effects of noise and bias correction. In simulations without noise and with the simpler, non‐bias‐corrected expression for the lower bound (not shown), the lower‐bound estimates of $\hat{a}$ are completely independent of a, as expected from the mathematical argument given above.

We conclude that the authors' formulas appear to work well because they lead to plausible bounds on the ascertainment ratio (≈0.2–0.5) for realistic values of the epidemic growth rate, but that they are in fact nearly unrelated to the true ascertainment ratio and should not be applied to estimate ascertainment ratios from disease outbreak incidence data.

## AUTHOR CONTRIBUTIONS

All authors contributed to the conceptual development of the paper. Michael Li and Benjamin M. Bolker wrote computer code for simulations and figures. Benjamin M. Bolker wrote the first draft of the paper. All authors commented and edited to produce the final version.

## CONFLICT OF INTEREST

The authors declare no conflict of interest.

## Data Availability

No data are used in the paper; all code for simulations is available at https://doi.org/10.5281/zenodo.7473422.
